# Reproductive outcomes following contraceptive discontinuation for method-related reasons: An analysis of 49 Demographic and Health Surveys

**DOI:** 10.1371/journal.pgph.0002143

**Published:** 2023-11-08

**Authors:** Alison Gemmill, Dana Sarnak, Sarah E. K. Bradley, Eve Brecker, Kaitlyn Patierno

**Affiliations:** 1 Department of Population, Family and Reproductive Health, Johns Hopkins Bloomberg School of Public Health, Baltimore, Maryland, United States of America; 2 Abt Associates, Bethesda, Maryland, United States of America; 3 Population Reference Bureau, Washington, District of Columbia, United States of America; UNC Gillings School of Global Public Health: The University of North Carolina at Chapel Hill Gillings School of Global Public Health, UNITED STATES

## Abstract

Contraceptive discontinuation for method-related reasons is a common experience in low- and middle-income countries (LMICs) and may heighten women’s risk of unintended pregnancy. Few studies have provided a comprehensive assessment of reproductive outcomes following contraceptive discontinuation in LMICs over the last decade. Using cross-sectional data from 49 Demographic and Health Surveys, we applied competing risks estimation to calculate monthly probabilities of contraceptive resumption and pregnancy over a 12-month period among pooled, regional, and country-specific samples of women who discontinued contraception for method-related reasons (corresponding to 174,726 episodes of discontinuation). We also examined the pregnancy intention status of births/current pregnancies among those who became pregnant in the 12 months following contraceptive discontinuation for method-related reasons. In the pooled sample, the three-month probability of pregnancy and resumption of contraceptive use was 12% and 47%, respectively; by 12 months these probabilities increased to 22% and 55%, respectively. Country-specific analyses show that the probabilities of resuming contraception by three months ranged from 15% in Liberia, Mali, and Sierra Leone to 85% in Bangladesh. On average, the majority of pregnancies/births that occured following discontinuation for method-related reasons were subsequently reported as unintended. However, reports varied depending on when women became pregnant within the 12 months following discontinuation. Our findings suggest the need for more nuanced measures of contraceptive use dynamics—including measures that distinguish between women who resume use of contraception from women who remain at risk of pregnancy in the short period after discontinuation—to better inform specific policies and interventions, particularly aimed at those who remain at risk of pregnancy.

## Introduction

Contraceptive discontinuation among women who want to avoid pregnancy is increasingly the focus of research intended to develop interventions and programs that can better meet clients’ family planning needs [1]. Women discontinue contraceptive use for many reasons. For example, some women discontinue because they want to become pregnant; others discontinue due to “method-related reasons” (e.g., side effects, lack of access, or inconvenience). Historically, researchers have assumed that women who discontinued contraception due to method-related reasons were at risk of an unintended pregnancy. Early seminal studies from select countries support this assumption, finding high proportions of unwanted births occurring soon after contraceptive discontinuation [2, 3]. More recently, Jain and Winfrey [4] estimated that contraceptive discontinuation may account for about one-third of unintended births in low- and middle- income countries (LMICs). Therefore, understanding reproductive behaviors and outcomes following discontinuation remains an important research priority.

Only one publication to date (Ali et al.) has examined the reproductive outcomes following discontinuation on a global scale [5]. This report included analyses of women’s reproductive outcomes three months after discontinuation in 17 countries across specific methods, with a focus on women presumably in need of contraception (i.e., those who discontinued for method-related reasons). They found that women’s reproductive outcomes varied greatly across countries; for example, while 80% of women had switched to another method three months following discontinuation in Morocco, Moldova, Turkey, and Vietnam, the probabilities of switching in sub-Saharan African countries were much lower [5]. Similar country variations also existed in the proportion of women who became pregnant within three months following discontinuation, ranging from 3% in Peru to 20% in the Dominican Republic [5].

Ali et al. further examined the reproductive outcomes of women 12 months after discontinuation to assess the pregnancy intention status of any subsequent live birth or pregnancy at the time of the survey and found wide variations in the proportion reporting the pregnancy to be unintended [5]. For example, while the probability of experiencing a pregnancy was highest in Malawi (37%), two thirds of those pregnancies were reported as unintended. This is in stark contrast to Peru, where the probability of pregnancy was only 7%, of which 80% were reported as unintended. Although the authors compared the pregnancy intention status of subsequent pregnancies and births following discontinuation between modern and traditional methods, it remains unclear whether there were differences in pregnancy intention status by type of method discontinued, as well as whether the pregnancy intention status of the reported pregnancy or birth varied by the time since discontinuation.

Since Ali et al.’s seminal report [5], several studies have brought attention to key factors that should be considered when measuring the risk of unintended pregnancy in women after discontinuation. First, while a shorter duration between discontinuation and method adoption should reduce women’s risk of unintended pregnancy, the literature uses inconsistent definitions of “timely” method switching. Indeed, some sources define method switching as occurring between one to three months after discontinuation [6–9]; others use longer intervals [10, 11]. Few studies have examined monthly patterns of contraceptive use following discontinuation to inform optimal measures of contraceptive switching.

Second, the historical emphasis on measuring *switching* to other methods, and especially more effective methods, ignores the possibility that women can reduce their risk of unintended pregnancy by *resuming* any recognized contraceptive method. Thus, a more nuanced understanding of the probabilities of pregnancy and contraceptive use following contraceptive discontinuation is warranted. Finally, growing evidence regarding the evolving nature of fertility intentions indicates that while many women desire to avoid pregnancy immediately after discontinuation, these preferences can change even over short periods of time [12].

The overall aim of present study is to update and expand existing the research by using recent Demographic and Health Survey (DHS) data, providing a more detailed picture of reproductive outcomes following discontinuation, and including a more nuanced analysis of retrospective pregnancy desires. To accomplish this aim, our objectives were as follows: (1) to quantify cumulative monthly probabilities of contraceptive resumption and pregnancy over a 12-month period, using contraceptive nonuse as a competing risk, and (2) to compare the pregnancy intention status of births and current pregnancies among those who became pregnant in the 12 months after contraceptive discontinuation, and to examine whether this status varies by time since discontinuation. We conducted all analyses are conducted separately using pooled, regional, and country-specific samples, as well as by the specific method discontinued, making this the most comprehensive and up-to-date analysis on the topic.

## Methods

We used cross-sectional data from 49 DHS conducted after 2009. The DHS are nationally representative household surveys conducted approximately every five years that collect data on key population and health indicators. For countries with more than one survey during this period, we used the most recent survey. Table A in S1 Text displays a list of all 49 surveys, regional classifications, and the corresponding sample sizes included in our analysis.

Our analysis specifically used the DHS reproductive calendar module, which retrospectively collects monthly data on contraceptive use and pregnancies in the five years prior to the interview. In any month for which a woman reported discontinuation of a contraceptive method, she also reported the reason for discontinuation. Because women could use and discontinue contraception multiple times over the five-year period, our unit of analysis was an episode of discontinuation rather than an individual woman. We limited our study to contraceptive episodes that were discontinued for method-related reasons. These method-related reasons included the following: side effects/health concerns, lack of access/too far, costs too much, wanted a more effective method, inconvenient to use, and other method-related reasons to that were collected in select countries. We excluded episodes that were discontinued for any other reason, such as: women wanted to become pregnant, contraceptive failure, felt fatalistic, difficult to get pregnant/menopausal, marital dissolution, infrequent sex/husband away, husband disapproved, Ramadan, “other reasons,” don’t know, or missing.

We further limited our analysis to episodes of discontinuation that occurred 3–62 months prior to the interview date. This time frame, which is standardized across surveys with different calendar lengths, is the recommended period of observation to study contraceptive discontinuation using DHS data [13]. The three months immediately preceding the interview were excluded to avoid underestimating pregnancies that were not yet recognized at the time of the interview. Our final sample size for analysis across combined surveys was 174,726 episodes of discontinuation, with country-specific samples ranging from 220 episodes in Comoros to 39,257 episodes in India.

We used two different approaches to understand outcomes after contraceptive discontinuation among women who wanted to avoid pregnancy. First, we used competing risks estimation to separately quantify the cumulative probability of the following: (1) resumption of contraceptive use, and (2) pregnancy over a 12-month period, with nonuse of contraception as an additional competing risk. As noted above, we chose to focus on resumption of contraceptive use as an outcome, rather than switching to a different method, to capture all instances of contraceptive use following discontinuation.

We calculated month-specific probabilities using the *stcompet* command in Stata [14]. This method ensures that the cumulative incidence depends on the hazards associated with all competing events, rather than exclusively on the hazard specific to the event in question. Further documentation of the method can be found in Coviello and Boggess (2004) [14].

We defined resumption of contraceptive use as the first month of a subsequent episode of any contraceptive use following the month of discontinuation. We measured pregnancy as the first month of pregnancy reported in the contraceptive calendar, regardless of the outcome of the pregnancy. (Pregnancy terminations are recorded in the contraceptive calendar, but for most countries, we could not distinguish between spontaneous and induced abortions).

In our second approach, we examined the pregnancy intention status of live births (hereafter referred to as births) and pregnancies among those who became pregnant in the 12 months following contraceptive discontinuation for method-related reasons. Women were asked at the time of the survey to report pregnancy intentions of all births and any current pregnancies. Responses included whether, at the time of pregnancy, the birth/current pregnancy was wanted then, wanted later, or not wanted at all. We then linked these responses to births recorded in the contraceptive calendar, as well as to current pregnancies at the time of the survey (the survey does not collect the pregnancy intention status of previous pregnancies that do not end in live birth). For each country and region, we calculated the distribution of births/current pregnancies that were wanted then, wanted later, or not wanted at all, and defined unintended pregnancies as those that were either wanted later or not wanted at all.

To evaluate whether the distribution of the pregnancy intention status of births/current pregnancies after contraceptive discontinuation differed from the pregnancy intention status distribution in the general population, we compared the pregnancy intention status of births in our analytic sample to the pregnancy intentioon status of all the most recent births among all survey respondents (i.e., not just among those who discontinued contraceptive methods).

For both analytical approaches, we conducted analyses for all methods combined and separately by method type. The specific contraceptive methods we examined included: pill, IUD, injectables, implant, male condom, periodic abstinence, withdrawal, lactational amenorrhea method (LAM), and emergency contraception (EC). These methods are typically studied in the contrtaceptive discontinuation literature [8, 15]. Other contraceptive methods that did not fall into one of these method categories were included in the “all methods” group because there were too few observations to analyze those methods separately.

We conducted analyses separately for all countries combined, by region, and for each country. We used the following regional groupings: East and South Africa; West and Middle Africa; North Africa, West Asia, and Europe; Central, South Asia, Southeast Asia, and Oceania; and Latin America and the Caribbean. (See Table A in S1 Text for each country’s regional assignment). We did not include countries with fewer than 350 observations in the country-specific analyses. Table B in S1 Text presents the number of observations for each region and by method discontinued.

Finally, because women may have changed their fertility preferences in the 12-month period following discontinuation, we conducted a secondary analysis to explore potential variations in the pregnancy intention status responses as a function of time since discontinuation. As such, we compared the reported pregnancy intention status of births/pregnancies among those who became pregnant within three months of discontinuation with those who became pregnant six to 12 months following discontinuation.

To ensure equal contributions from the pooled analysis, we adjusted the survey weights provided by the DHS by applying a country-specific constant. This adjustment ensured that the sample of women from each of the 49 countries accounted for an equal 1/49 portion of the pooled sample so that the results were not driven by a single country with a large sample size. More details on this adjustment can be found in Bradley and Shiras (2022) [16]. We conducted all analyses in Stata 17, using the *svy* suite of commands.

### Ethics statement

Ethical approval was obtained by the institutions that administered the surveys and all analyses used anonymized databases. Patients or the public were not involved in the design, conduct, reporting, or dissemination plans of our research.

## Results

### Probabilities of resuming contraception or pregnancy following contraceptive discontinuation for method-related reasons

**Fig 1:**
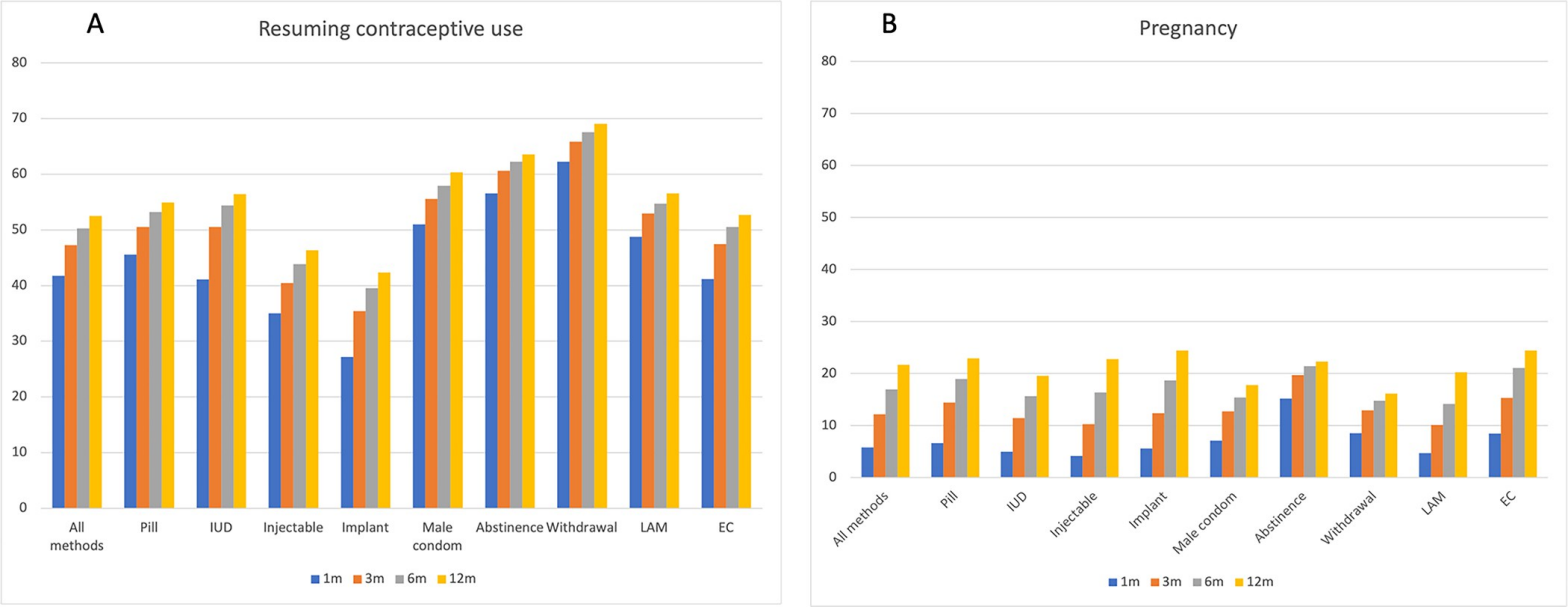
Month-specific cumulative probability of adopting another method [A] or pregnancy/birth [B] following contraceptive discontinuation among women who [still] want to avoid pregnancy.

Table 1 presents the month-specific cumulative probability of resuming contraception or pregnancy after contraceptive discontinuation for method-related reasons, for all methods, and by specific methods. These data are also represented in Fig 1A and 1B, which display cumulative probabilities of resuming contraception or pregnancy at one month, three months, six months, and 12 months.

**Table 1:** Month-specific cumulative probability of resuming contraceptive use or experiencing pregnancy following contraceptive discontinuation among women who discontinued for method-related reasons.

Method	Outcome	Month 1	Month 2	Month 3	Month 4	Month 5	Month 6	Month 7	Month 8	Month 9	Month 10	Month 11	Month 12
All methods	Resume any method	41.8	45.3	47.3	48.7	49.6	50.3	50.9	51.3	51.6	51.9	52.2	52.5
	Pregnancy	5.8	9.5	12.2	14.2	15.6	17.0	18.1	19.0	19.8	20.5	21.1	21.7
Pill	Resume any method	45.6	48.8	50.5	51.7	52.6	53.3	53.7	54.0	54.2	54.4	54.7	54.9
	Pregnancy	6.6	11.5	14.5	16.5	17.8	18.9	20.0	20.7	21.3	21.9	22.4	23.0
IUD	Resume any method	41.1	47.1	50.5	52.1	53.4	54.4	55.1	55.4	55.8	55.9	56.2	56.4
	Pregnancy	5.0	8.8	11.4	13.4	14.5	15.7	16.8	17.6	18.4	18.9	19.2	19.6
Injectables	Resume any method	35.0	38.4	40.5	42.3	43.2	43.9	44.7	45.1	45.5	45.8	46.1	46.4
	Pregnancy	4.2	7.4	10.3	12.9	14.7	16.4	17.9	19.1	20.2	21.1	22.0	22.8
Implants	Resume any method	27.2	32.3	35.4	37.3	38.8	39.5	40.3	40.6	41.1	41.7	42.0	42.4
	Pregnancy	5.6	9.4	12.4	14.9	17.0	18.7	20.1	21.1	21.9	22.8	23.7	24.4
Male condom	Resume any method	51.0	53.6	55.6	56.6	57.4	58.0	58.5	58.9	59.4	59.7	60.0	60.4
	Pregnancy	7.1	10.7	12.8	13.9	14.7	15.4	16.0	16.5	17.0	17.3	17.6	17.8
Periodic abstinence	Resume any method	56.6	59.1	60.6	61.5	62.0	62.3	62.5	62.8	63.0	63.1	63.2	63.6
	Pregnancy	15.2	18.4	19.7	20.6	21.3	21.5	21.7	21.9	22.1	22.2	22.3	22.3
Withdrawal	Resume any method	62.3	64.5	65.9	66.7	67.2	67.5	67.9	68.1	68.3	68.6	68.9	69.1
	Pregnancy	8.5	11.4	12.9	13.8	14.2	14.8	15.1	15.5	15.6	15.8	16.0	16.2
LAM	Resume any method	48.8	51.9	52.9	53.6	54.1	54.7	55.3	55.6	55.7	56.0	56.3	56.6
	Pregnancy	4.7	8.5	10.1	11.6	12.8	14.2	15.4	16.7	17.6	18.3	19.3	20.3
EC	Resume any method	41.2	45.3	47.4	48.4	49.5	50.5	51.1	51.3	51.8	52.1	52.6	52.7
	Pregnancy	8.5	12.0	15.3	17.5	19.4	21.1	22.3	23.3	23.8	24.1	24.3	24.4

For all methods and months, the monthly probability of pregnancy was lower than the probability of resuming contraception. The monthly probabilities displayed in Table 1 show that the increasing incidence of resuming contraception was highest within the first 3–4 months after discontinuation for method-related reasons. In other words, resuming contraceptive use was most prevalent in the first few months after discontinuation, with the largest increase occurring in the month immediately following discontinuation.

Beyond the three-month period following discontinuation, there were continued increases in the cumulative incidence of both outcomes, albeit in smaller increments. For example, among women who discontinued any contraceptive method for method-related reasons, the three-month probability of resuming contraceptive use and pregnancy following discontinuation was 47% and 12%, respectively. By six months, these probabilities for resuming contraceptive use and pregnancy increased to 50% and 16%, respectively, and by 12 months, these probabilities increased to 55% and 22%, respectively.

There were differences in probabilities by the methods that were discontinued (Table 1 and Fig 1A and 1B). The three-month probability of resuming contraception was highest for those who discontinued withdrawal (66%), periodic abstinence (61%), and the male condom (56%), but lowest for those who discontinued implants (35%) and injectables (41%). The three-month probability of pregnancy was highest among those who discontinued periodic abstinence (20%), EC (15%), and the pill (15%), but lowest for those who discontinued injectables (10%), LAM (10%), and the IUD (11%).

**Fig 2:**
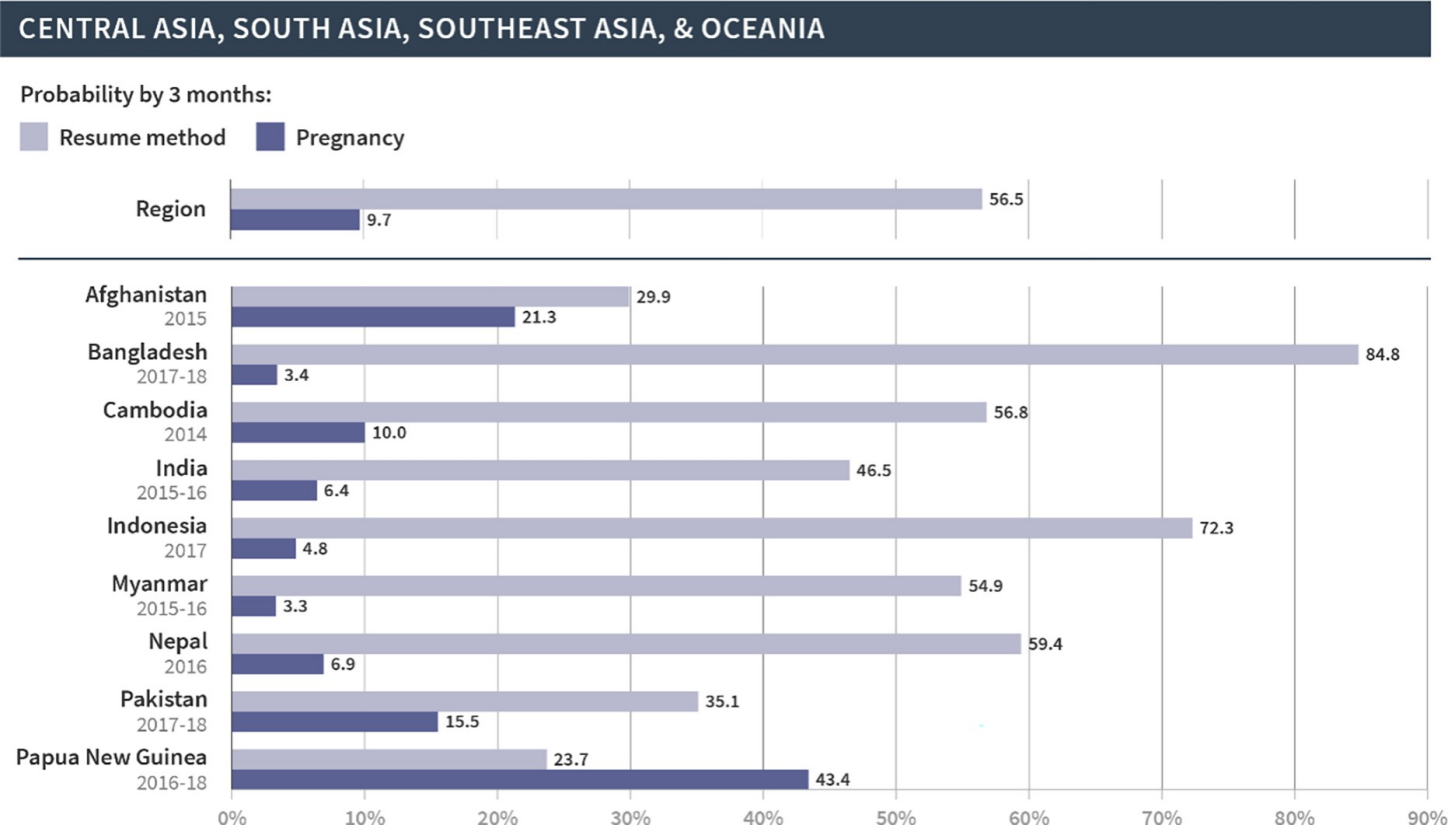
Region- and country-specific 3-month probabilities of resuming any contraceptive method and pregnancy in Central Asia, South Asia, Southeast Asia, and Oceania.

**Fig 3:**
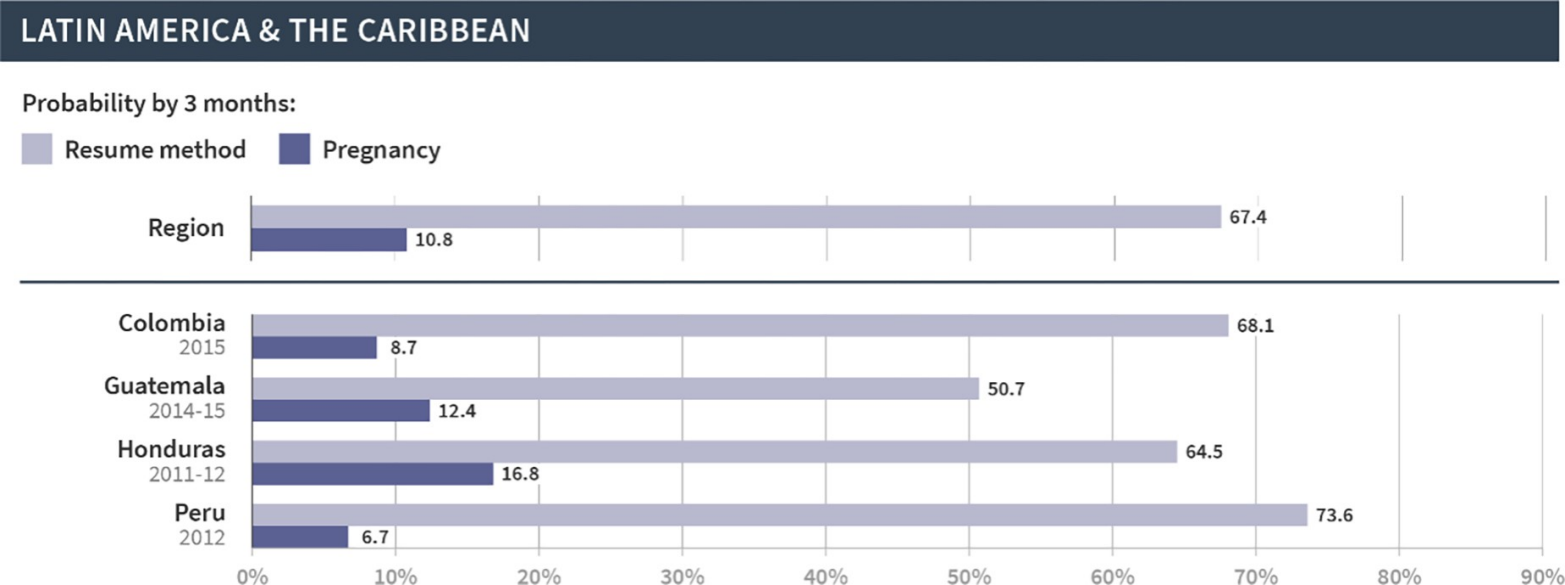
Region- and country-specific 3-month probabilities of resuming any contraceptive method and pregnancy in Latin America and the Caribbean.

**Fig 4:**
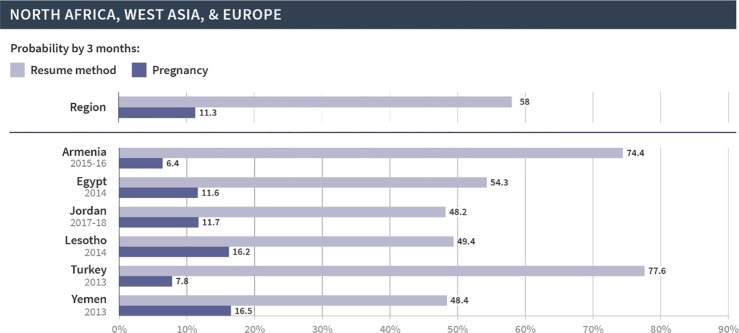
Region- and country-specific 3-month probabilities of resuming any contraceptive method and pregnancy in North Africa, West Asia, and Europe.

**Fig 5:**
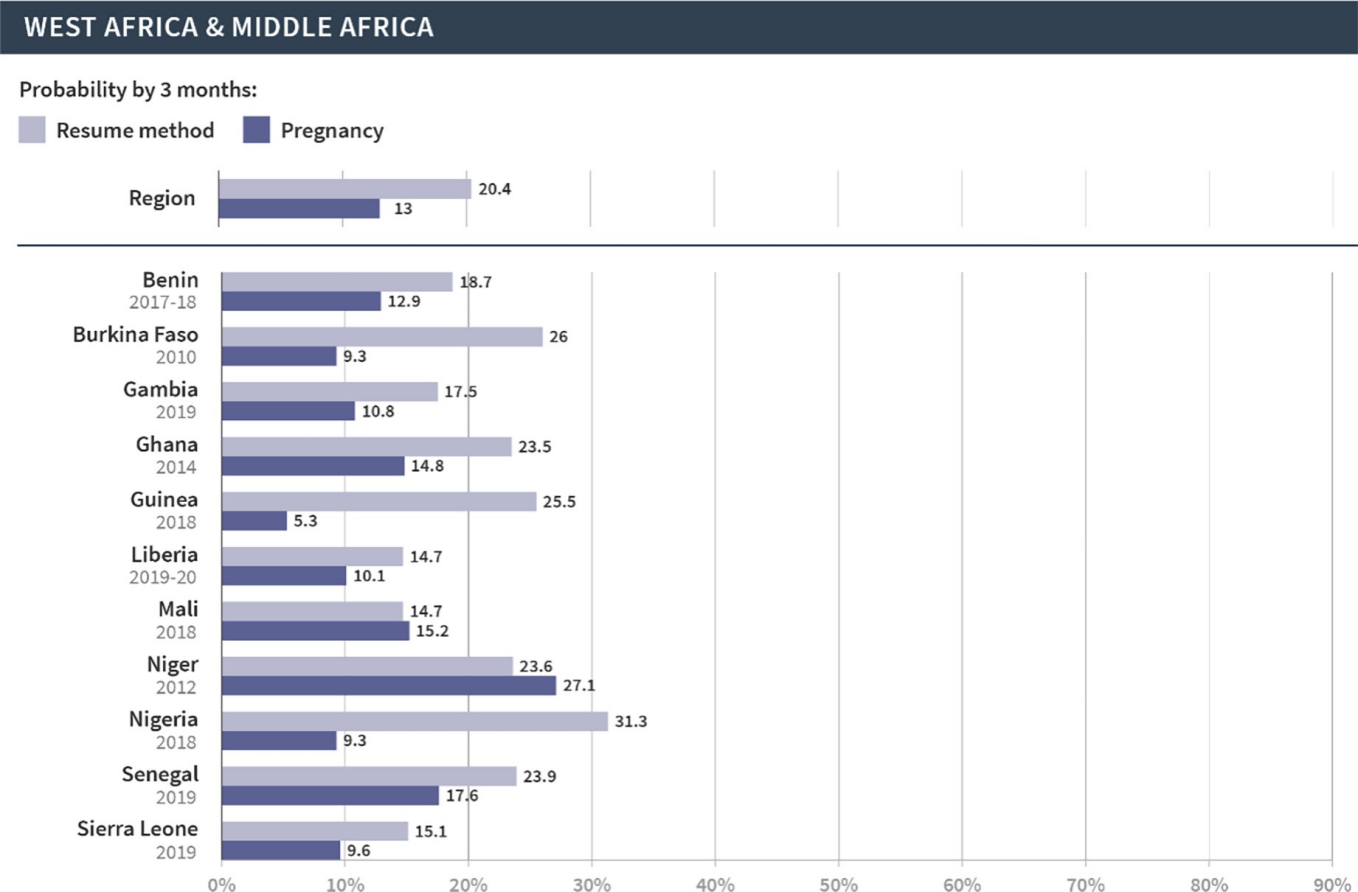
Region- and country-specific 3-month probabilities of resuming any contraceptive method and pregnancy in West Africa and Middle Africa.

**Fig 6:**
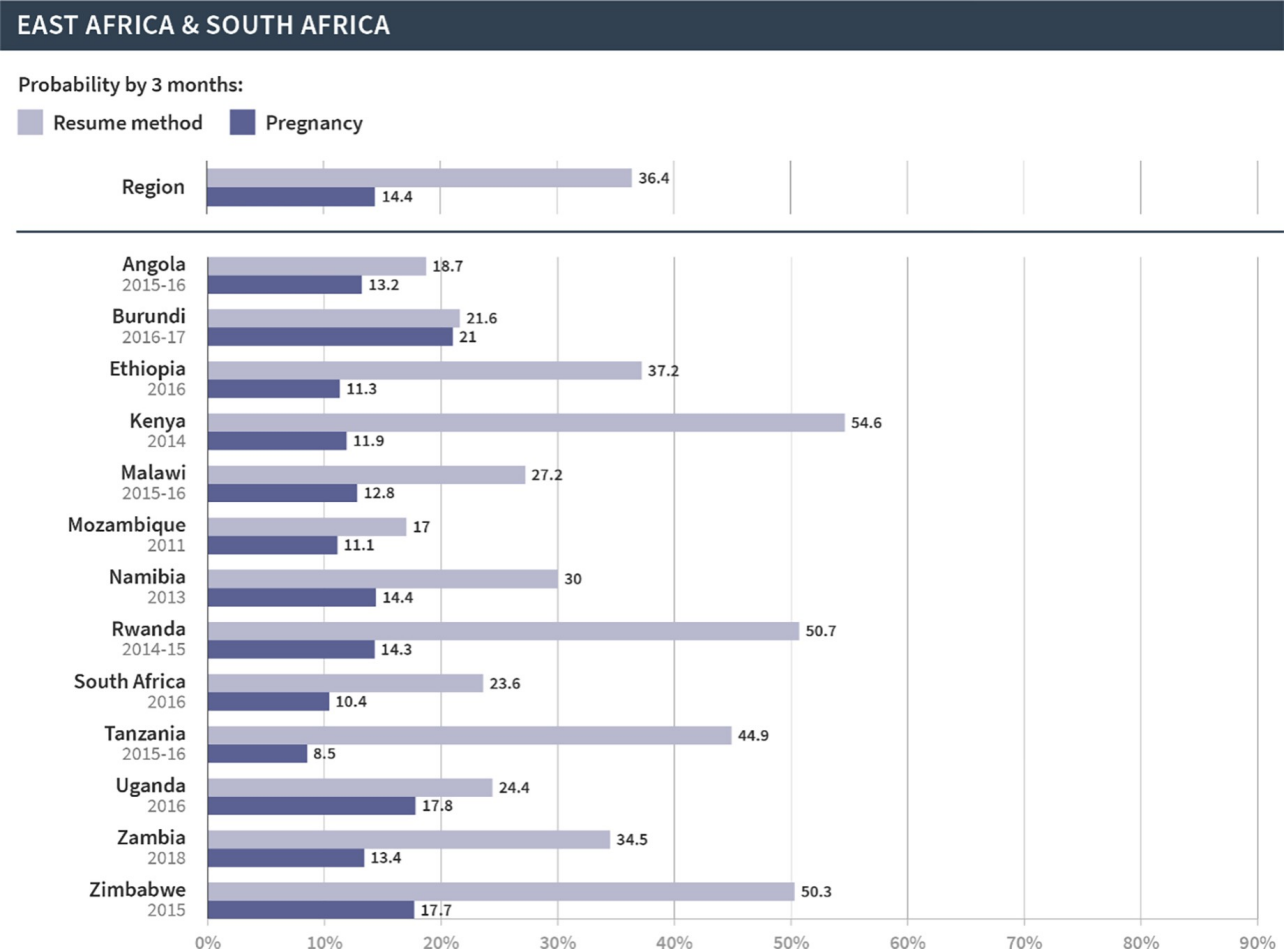
Region- and country-specific 3-month probabilities of resuming any contraceptive method and pregnancy in East Africa and Southern Africa.

We then focused on the regional and country-specific variations in the cumulative probability of resuming contraception or pregnancy at three months, since that time frame appears to capture key patterns in the reproductive outcomes following discontinuation. As shown in Figs 2–6, which are organized by region, the probabilities of pregnancy and the resumption of contraception following discontinuation mask the substantial variations at both the regional and country levels. First, at the regional level, at least half of women resumed a method by three months following discontinuation in Latin America and the Caribbean (67%), North Africa, West Asia and Europe (58%), and Central Asia, South Asia, Southeast Asia, and Oceania (57%). On the other hand, just over one third (36%) of women in East and South Africa and one fifth (20%) of women in West and Middle Africa resumed a method in the same period. The probability of pregnancy was more similar across regions and ranged from 10% in Central Asia, South Asia, Southeast Asia, and Oceania to 14% in East and South Africa.

We saw even wider variations at the country level. In Armenia, Bangladesh, Indonesia, Peru, and Turkey, over 70% of women resumed contraception within three months following discontinuation due to method-related reasons. On the other hand, less than 20% of women in Angola, Benin, The Gambia, Liberia, Maldives, Mozambique, Mali, and Sierra Leone resumed contraception during this period. Substantial variations across countries were also evident in the probability of pregnancy within three months after discontinuation of contraception. Less than 5% of women became pregnant following discontinuation in Bangladesh, Indonesia, and Myanmar; over 20% became pregnant in Afghanistan, Burundi, Niger, and Papua New Guinea. Finally, the probability of resuming contraception by three months was larger than the probability of pregnancy for all countries except in Papua New Guinea, Mali, and Niger. We did not investigate method-specific patterns in each country due to limited sample sizes.

### Pregnancy intention status of births and current pregnancies following contraceptive discontinuation for method-related reasons

Table 2 presents the pregnancy intention status of births and current pregnancies that resulted from pregnancies occurring in the 12 months following contraceptive discontinuation for method-related reasons. For comparison, Table 2 also shows the pregnancy intention status of all of the most recent births among the general population of reproductive-aged women. Results are presented for all regions combined (the pooled sample), as well as for each region. Across the entire sample, among the episodes of discontinuation that were followed by pregnancy in the 12-month period, 45% of births/current pregnancies were reported as being wanted then, 37% as wanted later, and 19% as not wanted at all. This pregnancy intention status distribution contrasts sharply with the distribution among all of the most recent births reported in the general population, wherein about 70% of births were wanted then, 21% were wanted later, and only 9% were not wanted at all.

**Table 2:** Comparison of pregnancy intention status of births/current pregnancies among those that occur 12 months after discontinuation among women who [still] want to avoid pregnancy and all most recent births among all survey respondents, by region.

	Wanted then	Wanted later	Wanted no more
All regions			
Post-discontinuation	44.5	36.5	19.1
Most recent births	69.8	20.8	9.4
East & South Africa			
Post-discontinuation	37.5	43.7	18.8
Most recent births	59.1	30.2	10.7
West & Middle Africa			
Post-discontinuation	63.0	29.4	7.5
Most recent births	77.2	18.6	4.2
North Africa, West Asia, & Europe			
Post-discontinuation	48.9	25.4	25.6
Most recent births	76.1	13.7	10.3
Central, South, & Southeast Asia & Oceania			
Post-discontinuation	61.2	17.6	21.3
Most recent births	82.7	9.5	7.8
Latin American & the Caribbean			
Post-discontinuation	27.1	46.4	26.5
Most recent births	52.9	28.7	18.4

This pattern was seen across all regions; in general, births and current pregnancies that occurred following discontinuation were more likely to be unintended compared to all of the most recent births/pregnancies in the general population. This difference was largest in North Africa, West Asia, and Europe, whereby only 49% of post-discontinuation births/pregnancies were reported as wanted then compared to 76% among the most recent births/pregnancies. The difference was smallest for West and Middle Africa; 63% of post-discontinuation births/pregnancies were wanted then compared to 77% among most recent births/pregnancies.

**Fig 7:**
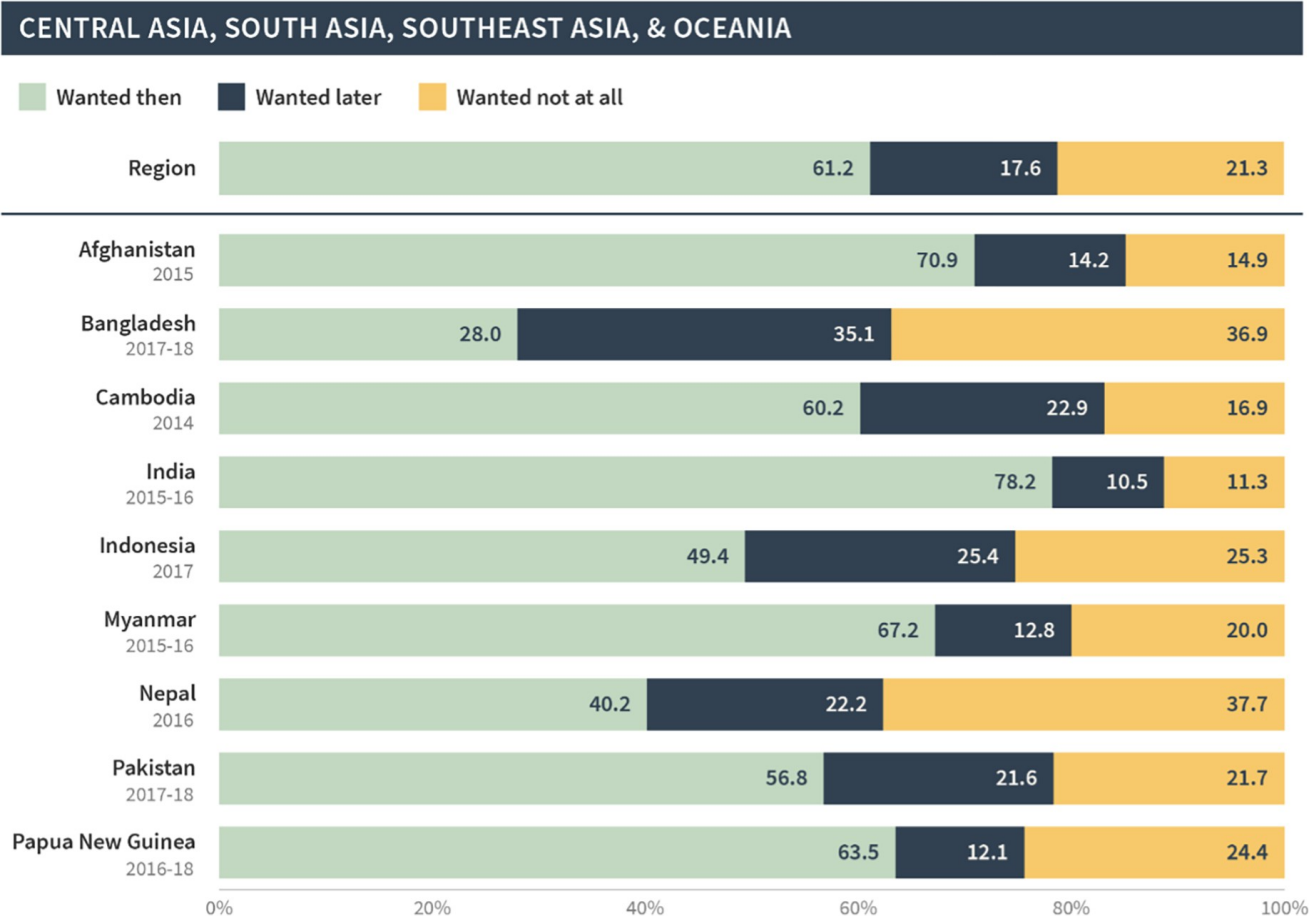
Distribution of pregnancy intention status of births (and current pregnancies) among pregnancies that occurred within 12 months of discontinuation among women who [still] want to avoid pregnancy in Central Asia, South Asia, Southeast Asia, and Oceania.

**Fig 8:**
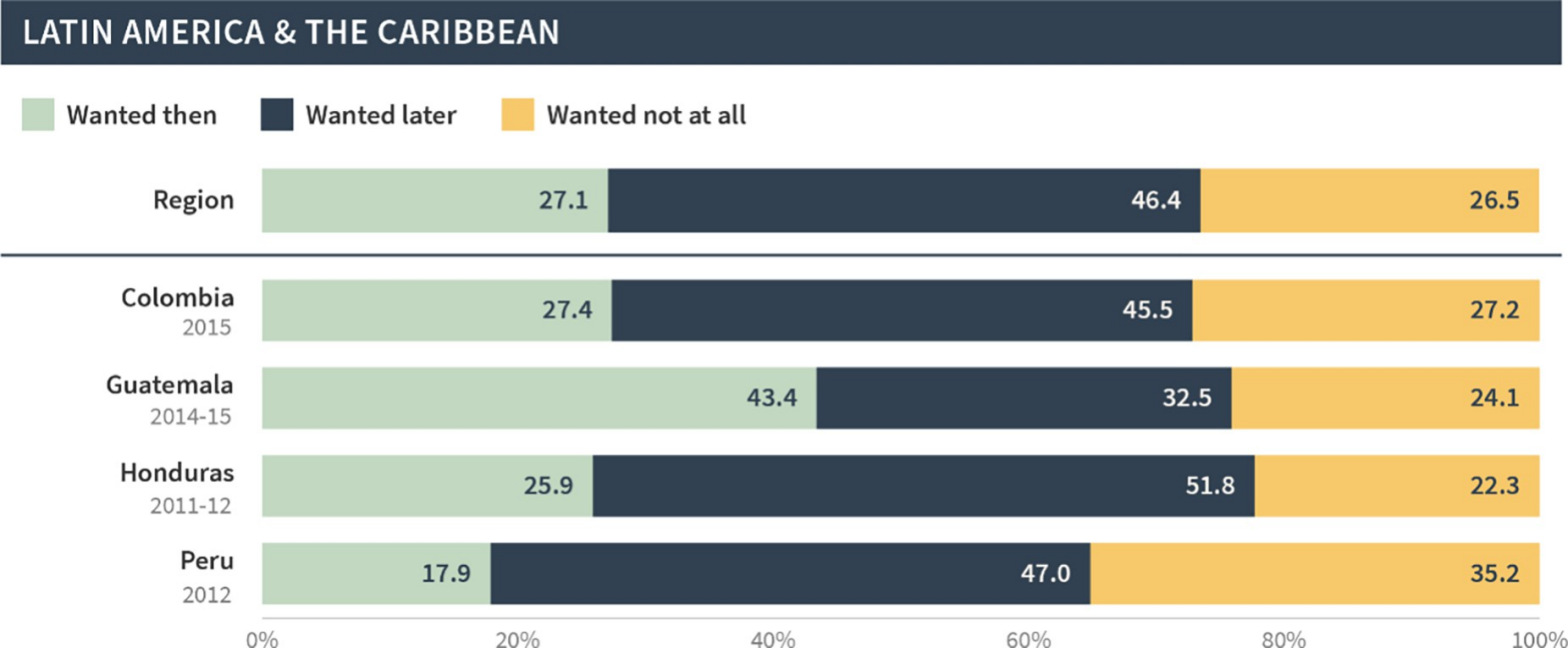
Distribution of pregnancy intention status of births (and current pregnancies) among pregnancies that occurred within 12 months of discontinuation among women who [still] want to avoid pregnancy in Latin America and the Caribbean.

**Fig 9:**
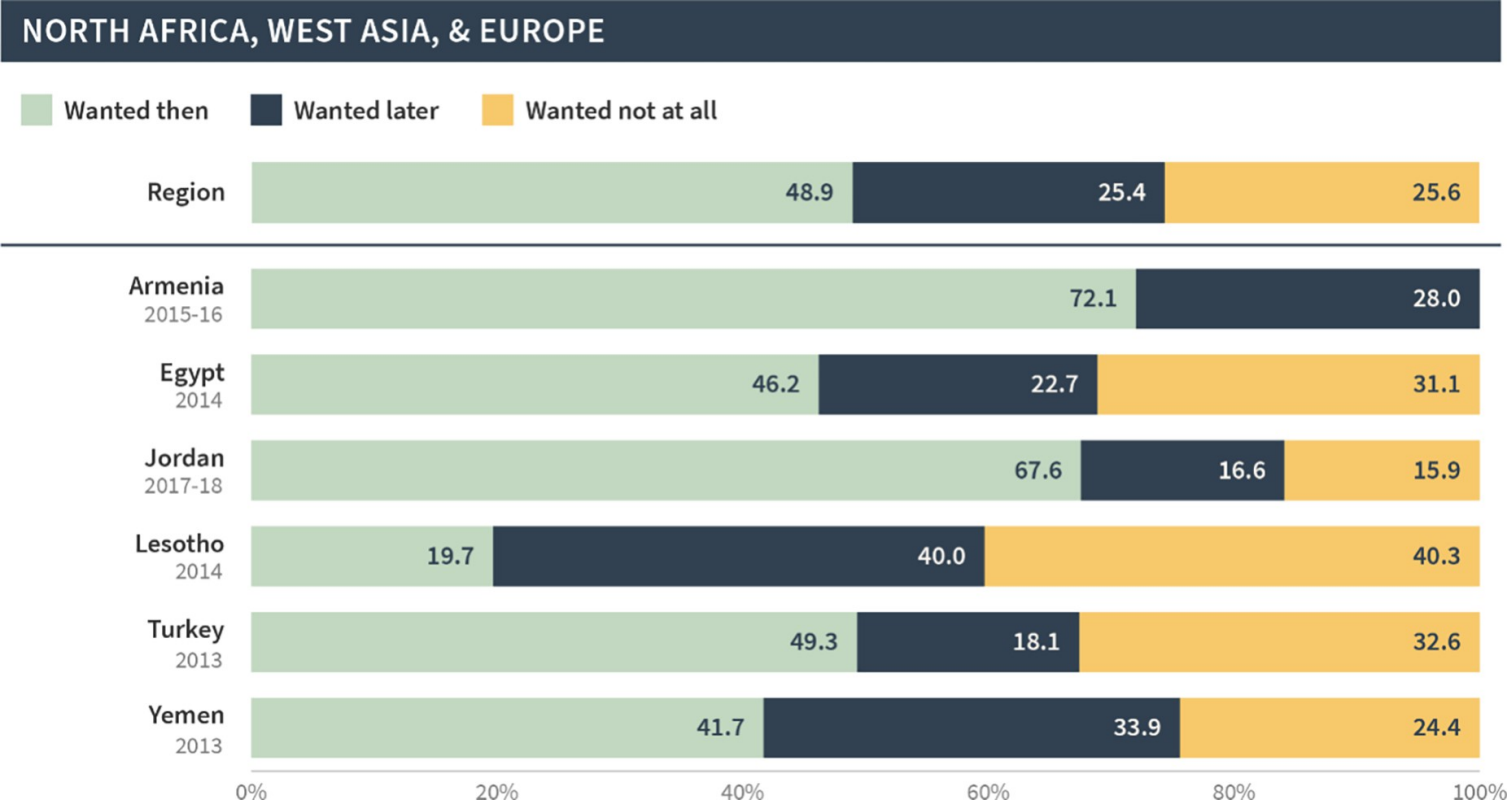
Distribution of pregnancy intention status of births (and current pregnancies) among pregnancies that occurred within 12 months of discontinuation among women who [still] want to avoid pregnancy in North Africa, West Asia, and Europe.

**Fig 10:**
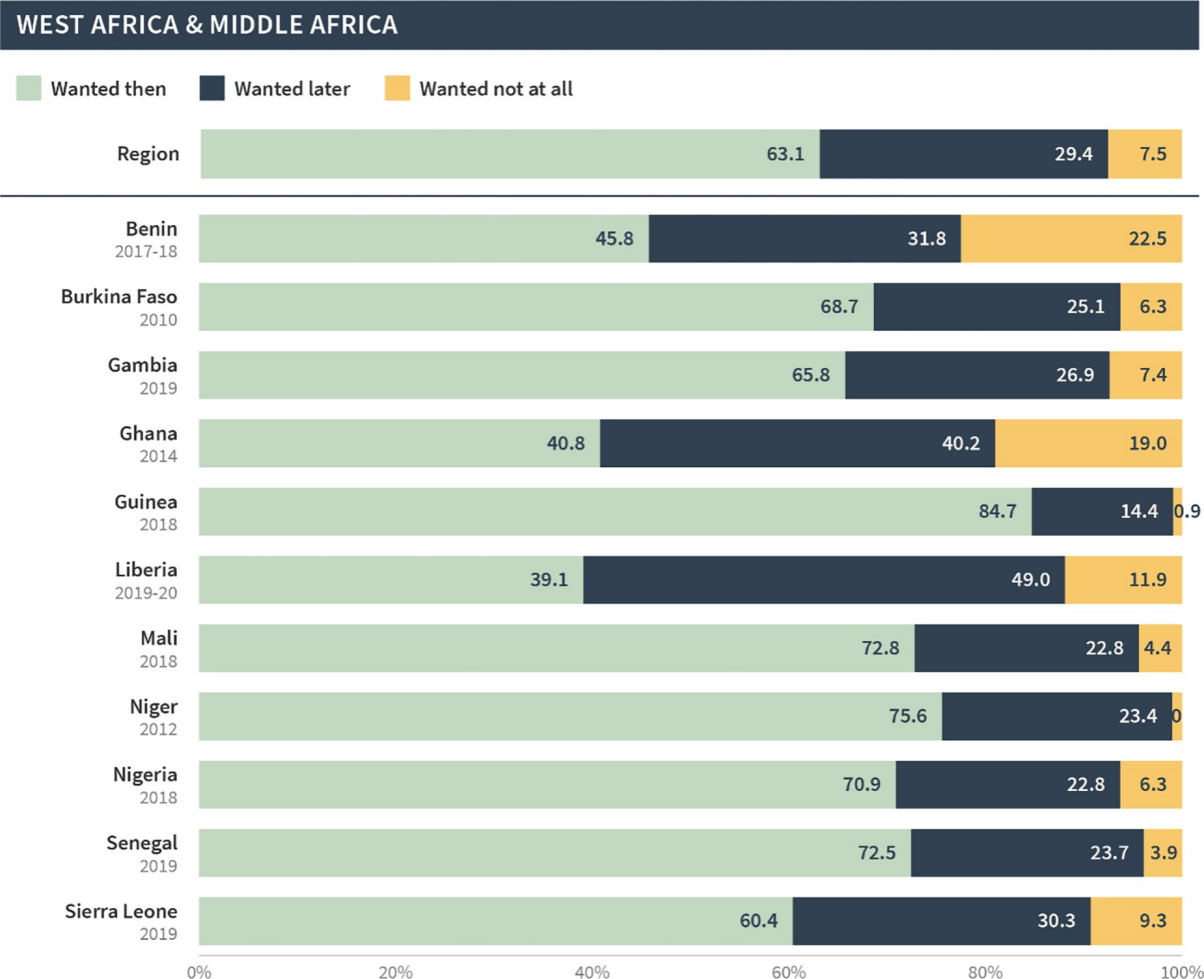
Distribution of pregnancy intention status of births (and current pregnancies) among pregnancies that occurred within 12 months of discontinuation among women who [still] want to avoid pregnancy in West Africa and Middle Africa.

**Fig 11:**
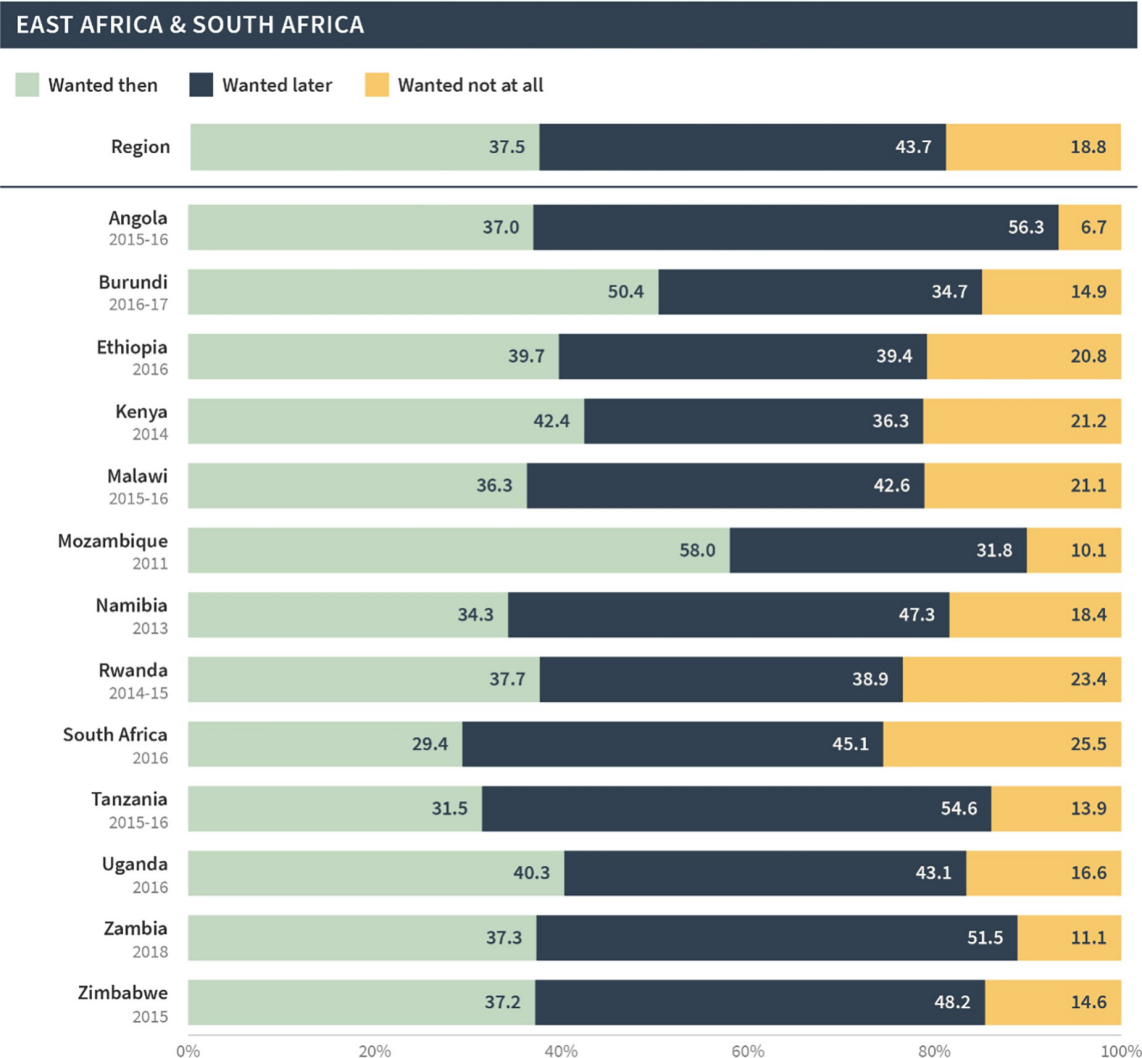
Distribution of pregnancy intention status of births (and current pregnancies) among pregnancies that occurred within 12 months of discontinuation among women who [still] want to avoid pregnancy in East Africa and Southern Africa.

As before, the global and regional pregnancy intention status distributions in our sample masked substantial variations at the country level. Figs 7–11 present the pregnancy intention status distribution for each region and country. We found that over 70% of births were classified as wanted then in Afghanistan, Armenia, Guinea, India, Mali, Niger, Nigeria and Senegal—even though these pregnancies began within three months of discontinuation due to a method-related reason. Further, one third or more of pregnancies/births in Bangladesh, Lesotho, Nepal, Peru, and Turkey were reported as not wanted at all.

The pregnancy intention status of births/pregnancies also differed according to the contraceptive method that was discontinued (Fig A in S1 Text). Unintended pregnancies were most prevalent among births and current pregnancies that occurred following discontinuation of the male condom (72%), injectables (58%), and the pill (57%). By contrast, unintended pregnancies were least common among users who discontinued LAM (31%), the IUD (41%), or the implant (42%). Although the distribution of the pregnancy intention status of births and current pregnancies differed by region, the *relationship* between the contraceptive method type that was discontinued and the pregnancy intention status was largely similar across regions, with some exceptions.

Figs B-F in S1 Text present the pregnancy intention status distribution for each region by method type. Similar to the overall pattern, across all regions, unintended pregnancies were most prevalent among births/pregnancies after discontinuation of male condoms, injectables, and the pill (with the exception of the pill in North Africa). In some regions, reports of unintended pregnancy were more common after contraceptive discontinuation of traditional methods such as withdrawal (Latin America and the Caribbean, West Africa, East Africa). Across all regions except North Africa, the proportion of pregnancies that were wanted then was highest after discontinuation of implants. Similarly, reports of wantedness after discontinuation of IUDs were high across all regions except East Africa. The prevalence of wanted births/pregnancies was also high after discontinuation of LAM in all regions except for Latin America and the Caribbean.

In secondary analyses using the pooled sample, the reported pregnancy intention status of births and current pregnancies was sensitive to the timing of when the pregnancy occurred following discontinuation. Fig G in S1 Text compares the proportion of births/pregnancies wanted then among those that were conceived within three months of discontinuation vs. those that were conceived 6–12 months following discontinuation. For all methods combined, the percent reported as wanted then was 40% for <3 months but increased to 51% for those pregnancies that occurred 6–12 months after discontinuation. When we compared the timing of responses by method type, we saw that for most methods, pregnancies that were conceived 6–12 months following discontinuation were more likely to be reported as wanted; however, we did not see this pattern for users who discontinued the IUD or the implant.

## Discussion

In our pooled sample from 49 DHS, we found that among women who discontinued contraception for method-related reasons, the monthly probability of resuming contraception was higher than the probability of pregnancy in the year after discontinuation. On average, close to half of the women who discontinued contraception due to method-related reasons resumed contraception within three months. Importantly, though, this varied widely across settings. Country-specific analyses showed that the probabilities of resuming contraception by three months ranged from 15% in Liberia, Mali, and Sierra Leone to 85% in Bangladesh. Therefore, our findings demonstrate that a substantial number of women did not resume contraception and continued to be at potential risk of unintended pregnancy following discontinuation.

In our pooled and regional samples, we found that the probabilities of resuming contraception varied by the type of method that was discontinued, with the highest probabilities for those who discontinued withdrawal, periodic abstinence, and male condoms, but lowest for those who discontinued implants and injectables. Although we did not examine the reasons for these differences by method, we hypothesize a few potential drivers. Women using hormonal methods, such as implants and injectables, may be unhappy with the side effects―including contraceptive-induced menstrual changes―and therefore less eager to resume another method [11, 17–20]. It may also be more challenging for women using methods that require active discontinuation to resume a method, particularly if they experience provider bias [21]. A potential area of future research is to examine country case studies where resumption of contraception is highest to see why some women effectively resume contraception quickly while others do not.

As in prior studies [5], our study shows that on average, the majority of pregnancies/births that occur after discontinuation for method-related reasons are subsequently reported as unintended. However, we also found widespread variations in the pregnancy intention status distribution across regions and countries, which largely paralleled the pregnancy intention status of births in the general population. A unique contribution of our analysis was comparing the pregnancy intention status of births/pregnancies by time since discontinuation (3 vs. 6–12 months). As expected, the probabilities of reporting wanting the pregnancy “then” were higher when the pregnancy occurred 6–12 months following discontinuation vs. three months, most likely due to a post hoc rationalization bias that has been discussed at length in other studies [12, 22], as well as women’s changing intentions and acceptability of pregnancy over time [12, 23].

We found that the pregnancy intention status distribution differed by the method discontinued, with those using less effective and traditional methods (e.g., periodic abstinence, withdrawal, or LAM) more likely to report subsequent pregnancies or births as wanted then when compared to pill and injectable users. However, we also found that almost 60% of IUD and implant users—methods which require active discontinuation—reported their subsequent pregnancies/births as wanted then. Reasons for these patterns may be tied to variations in women’s motivation to avoid pregnancy [24], but we did not test this hypothesis because data on women’s desire to avoid a pregnancy at the time of discontinuation were not collected.

While many analyses of contraceptive switching have focused on switching to more effective methods [5, 25], we chose to document any resumption of contraceptive use as an outcome. Nevertheless, these findings inform ongoing discussion on the definition of method “switching,” since some sources define switching as occurring within one to three months of discontinuation; while other studies have examined longer time frames [10, 11]. Further, studies have not provided detailed information on the month-specific probabilities of outcomes following discontinuation that can inform discussions about how timely switching is defined. Our findings showed that the probability of resuming contraceptives (generally due to contraceptive switching) was most common in the first month following discontinuation, with some additional, albeit small, increases in the incidence in the next two to four months. After four months, however, the probability of contraceptive resumption appeared to plateau. Therefore, we recommend that switching be considered in a shorter timeframe to optimize the reduction in pregnancy exposure and to align with the timing when most women typically switch.

Despite the many strengths of the present study, there are some limitations to note. First, for most countries in our study, we could not determine whether reported terminations were the result of spontaneous or induced abortions, nor did we have data on the pregnancy intention status of these terminations. Second, because terminations are underreported in surveys [26, 27], we were not able to capture an important and programmatically relevant share of pregnancies that occurred after discontinuation. Third, we lacked detailed data on fertility intentions at the time of discontinuation, or in the post-discontinuation period. As noted earlier, short-term changes in pregnancy intention have been well-documented in several contexts [12], and pregnancy ambivalence is also common [28, 29]. Fourth, research has indicated some concerns regarding the quality of the data in the contraceptive calendar, especially with respect to recall bias [30, 31]. However, our inclusion of data from the full contraceptive calendar ensured adequate sample sizes for country-specific analyses. Fifth, the outcomes we studied were recorded as mutually exclusive categories in each month, which may have potentially underestimated instances when contraceptive resumption and failure occurred in the same month. Finally, our analyses used population-level data among all reproductive-aged women and did not disaggregate by characteristics that might have influenced patterns, including age, education, and urban/rural residence. Future research should therefore evaluate whether the patterns we described herein are similar among population subgroups, as such characteristics have been shown to shape contraceptive use, preferences, and access.

The present study highlights how global family planning metrics that focus on discontinuation rates alone, such Indicator 18a in the FP2020 Core Indicators [32], provide an incomplete picture of women’s contraceptive behaviors and may inform misguided strategies to enhance family planning efforts. While a substantial number of women may be at risk of unintended pregnancy following discontinuation, our results showed that that the majority of women in some countries and regions were able to resume contraception in a timely manner to avoid this outcome, corroborating several recent studies [33, 34]. We recommend the development of more nuanced measures of contraceptive use dynamics that distinguish women who resume use of contraception from those who remain at risk of pregnancy in the short period following discontinuation in order to better inform specific polices and interventions, particularly aimed at the latter group.

## Conclusion

Policy and program decisionmakers should continue focusing on interventions that meet the contraceptive needs of women who desire to avoid pregnancy, including measures that consider contraceptive discontinuation and resumption. Evidence on contraceptive use such as the present study supports decisionmakers in understanding the types of initiatives that can best serve clients, such as strengthening contraceptive counseling or ensuring the availability of a broad method mix. In this way, women may select or switch to the method that best works for their lifestyle and meets their preferences. Family planning policies, strategies, and program guidelines supporting such efforts are essential to enhancing contraceptive continuation among existing users who wish to prevent or space pregnancies.

## Supporting information

S1 Text **Table A**. List of surveys, analysis region, and number of observations. **Table B.** Unweighted Ns for analysis according to specific method and by region. **Fig A.** Distribution of planning status of births (and current pregnancies) among pregnancies that occurred within 12 months of discontinuation among women who [still] want to avoid pregnancy. **Fig B.** Distribution of planning status of births (and current pregnancies) among pregnancies that occurred within 12 months of discontinuation among women who [still] want to avoid pregnancy in Central Asia, South Asia, Southeast Asia, and Oceania. **Fig C.** Distribution of planning status of births (and current pregnancies) among pregnancies that occurred within 12 months of discontinuation among women who [still] want to avoid pregnancy in Latin America and the Caribbean. **Fig D.** Distribution of planning status of births (and current pregnancies) among pregnancies that occurred within 12 months of discontinuation among women who [still] want to avoid pregnancy in North Africa, West Asia, and Europe. **Fig E.** Distribution of planning status of births (and current pregnancies) among pregnancies that occurred within 12 months of discontinuation among women who [still] want to avoid pregnancy in West and Middle Africa. **Fig F.** Distribution of planning status of births (and current pregnancies) among pregnancies that occurred within 12 months of discontinuation among women who [still] want to avoid pregnancy in East and Southern Africa. **Fig G.** Comparison of the proportion of pregnancies wanted “then” among those that occur within 3 months of discontinuation vs. 6–12 months.
(DOCX)
