# Supplementary material for: Reproductive outcomes following contraceptive discontinuation for method-related reasons: An analysis of 49 Demographic and Health Surveys
Source: PLOS Glob Public Health. 2023 Nov 8;3(11):e0002143. doi: 10.1371/journal.pgph.0002143 (PMC10631694; doi:10.1371/journal.pgph.0002143)
Supplement: S1 Text — Table A. List of surveys, analysis region, and number of observations. Table B. Unweighted Ns for analysis according to specific method and by region. Fig A. Distribution of planning status of births (and current pregnancies) among pregnancies that occurred within 12 months of discontinuation among women who [still] want to avoid pregnancy. Fig B. Distribution of planning status of births (and current pregnancies) among pregnancies that occurred within 12 months of discontinuation among women who [still] want to avoid pregnancy in Central Asia, South Asia, Southeast Asia, and Oceania. Fig C. Distribution of planning status of births (and current pregnancies) among pregnancies that occurred within 12 months of discontinuation among women who [still] want to avoid pregnancy in Latin America and the Caribbean. Fig D. Distribution of planning status of births (and current pregnancies) among pregnancies that occurred within 12 months of discontinuation among women who [still] want to avoid pregnancy in North Africa, West Asia, and Europe. Fig E. Distribution of planning status of births (and current pregnancies) among pregnancies that occurred within 12 months of discontinuation among women who [still] want to avoid pregnancy in West and Middle Africa. Fig F. Distribution of planning status of births (and current pregnancies) among pregnancies that occurred within 12 months of discontinuation among women who [still] want to avoid pregnancy in East and Southern Africa. Fig G. Comparison of the proportion of pregnancies wanted “then” among those that occur within 3 months of discontinuation vs. 6–12 months. (DOCX) [file pgph.0002143.s001.docx]

**Table A.** List of surveys, analysis region, and number of observations.

| Survey | Analysis Region | Number of observations |
| --- | --- | --- |
| Afghanistan 2015 | Central/South/Southeast Asia/Oceania | 2,089 |
| Albania 2017-18 | North Africa/West Asia/Europe | 341 |
| Angola 2015-16 | East/South Africa | 589 |
| Armenia 2015-16 | North Africa/West Asia/Europe | 476 |
| Bangladesh 2017-18 | Central/South/Southeast Asia/Oceania | 6,169 |
| Benin 2017-18 | West/Middle Africa | 1,021 |
| Burkina Faso 2010 | West/Middle Africa | 780 |
| Burundi 2016-17 | East/South Africa | 2,201 |
| Cambodia 2014 | Central/South/Southeast Asia/Oceania | 2,186 |
| Colombia 2015 | Latin America and the Caribbean | 12,183 |
| Comoros 2012 | East/South Africa | 220 |
| Egypt 2014 | North Africa/West Asia/Europe | 5,167 |
| Ethiopia 2016 | East/South Africa | 1,853 |
| Gambia 2019 | West/Middle Africa | 1,091 |
| Ghana 2014 | West/Middle Africa | 577 |
| Guatemala 2014-15 | Latin America and the Caribbean | 4,208 |
| Guinea 2018 | West/Middle Africa | 1,208 |
| Honduras 2011-12 | Latin America and the Caribbean | 11,369 |
| India 2015-16 | Central/South/Southeast Asia/Oceania | 39,257 |
| Indonesia 2017 | Central/South/Southeast Asia/Oceania | 11,523 |
| Jordan 2017-18 | North Africa/West Asia/Europe | 1,890 |
| Kenya 2014 | East/South Africa | 3,270 |
| Kyrgyz Republic 2012 | Central/South/Southeast Asia/Oceania | 514 |
| Lesotho 2014 | North Africa/West Asia/Europe | 1,625 |
| Liberia 2019-20 | West/Middle Africa | 1,617 |
| Malawi 2015-16 | East/South Africa | 6,048 |
| Maldives 2016-17 | Central/South/Southeast Asia/Oceania | 326 |
| Mali 2018 | West/Middle Africa | 696 |
| Mozambique 2011 | East/South Africa | 1,060 |
| Myanmar 2015-16 | Central/South/Southeast Asia/Oceania | 2,396 |
| Namibia 2013 | East/South Africa | 2,100 |
| Nepal 2016 | Central/South/Southeast Asia/Oceania | 1,852 |
| Niger 2012 | West/Middle Africa | 1,350 |
| Nigeria 2018 | West/Middle Africa | 2,338 |
| Pakistan 2017-18 | Central/South/Southeast Asia/Oceania | 881 |
| Papua New Guinea 2016-18 | Central/South/Southeast Asia/Oceania | 1,455 |
| Peru 2012 | Latin America and the Caribbean | 15,028 |
| Rwanda 2014-15 | East/South Africa | 2,707 |
| Senegal 2019 | West/Middle Africa | 781 |
| Sierra Leone 2019 | West/Middle Africa | 1,482 |
| South Africa 2016 | East/South Africa | 1,064 |
| Tajikistan 2017 | Central/South/Southeast Asia/Oceania | 568 |
| Tanzania 2015-16 | East/South Africa | 2,085 |
| Timor-Leste 2016 | Central/South/Southeast Asia/Oceania | 500 |
| Turkey 2013 | North Africa/West Asia/Europe | 1,875 |
| Uganda 2016 | East/South Africa | 4,138 |
| Yemen 2013 | North Africa/West Asia/Europe | 5,117 |
| Zambia 2018 | East/South Africa | 3,204 |
| Zimbabwe 2015 | East/South Africa | 2,251 |
| **Total** |  | **174,726** |

**Table B.** Unweighted Ns for analysis according to specific method and by region

| Method | All regions | East/ South Africa | West/ Middle Africa | North Africa/ West Asia/ Europe | Central/ South/ Southeast Asia/ Oceania | Latin America and the Caribbean |
| --- | --- | --- | --- | --- | --- | --- |
| Pill | 42,118 | 6,521 | 2,660 | 4,701 | 19,840 | 8,396 |
| IUD | 10,046 | 524 | 398 | 3,007 | 4,412 | 1,705 |
| Injectables | 57,967 | 18,582 | 5,329 | 2,483 | 13,966 | 17,607 |
| Implants | 7,702 | 3,225 | 1,801 | 235 | 1,425 | 1,016 |
| Male condom | 26,076 | 3,527 | 677 | 713 | 14,296 | 6,863 |
| Periodic abstinence | 9,702 | 459 | 218 | 178 | 6,651 | 2,196 |
| Withdrawal | 12,685 | 750 | 338 | 1,364 | 7,185 | 3,048 |
| LAM | 5,420 | 444 | 1,158 | 1,804 | 1,496 | 518 |
| EC | 2,021 | 211 | 281 | 31 | 205 | 1,293 |
| Other modern | 622 | 172 | 81 | 27 | 216 | 126 |
| Other traditional | 367 | -- | -- | 323 | 24 | 20 |
| All methods | 174,726 | 34,415 | 12,941 | 14,866 | 69,716 | 42,788 |

**Fig A.** Distribution of planning status of births (and current pregnancies) among pregnancies that occurred within 12 months of discontinuation among women who [still] want to avoid pregnancy.


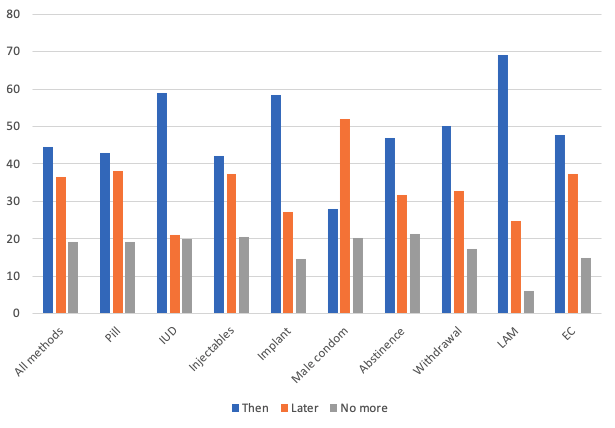


**Fig B.** Distribution of planning status of births (and current pregnancies) among pregnancies that occurred within 12 months of discontinuation among women who [still] want to avoid pregnancy in Central Asia, South Asia, Southeast Asia, and Oceania.

**Fig C.** Distribution of planning status of births (and current pregnancies) among pregnancies that occurred within 12 months of discontinuation among women who [still] want to avoid pregnancy in Latin America and the Caribbean.

**Fig D.** Distribution of planning status of births (and current pregnancies) among pregnancies that occurred within 12 months of discontinuation among women who [still] want to avoid pregnancy in North Africa, West Asia, and Europe.

**Fig E.** Distribution of planning status of births (and current pregnancies) among pregnancies that occurred within 12 months of discontinuation among women who [still] want to avoid pregnancy in West and Middle Africa.

**Fig F.** Distribution of planning status of births (and current pregnancies) among pregnancies that occurred within 12 months of discontinuation among women who [still] want to avoid pregnancy in East and Southern Africa.

**Fig G.** Comparison of the proportion of pregnancies wanted “then” among those that occur within 3 months of discontinuation vs. 6-12 months.

**
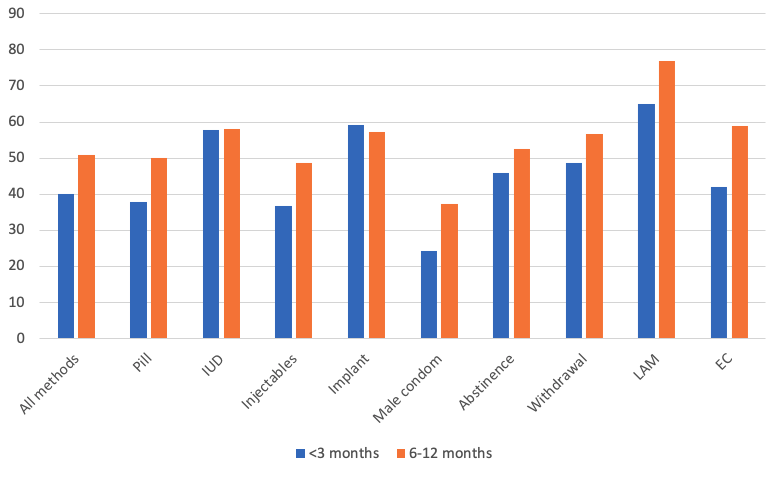
**
